# Development of a smartphone-based app to support the differential diagnosis in patients with primary left ventricular hypertrophy

**DOI:** 10.1093/ehjdh/ztaf105

**Published:** 2025-09-16

**Authors:** Niccolò Maurizi, Emanuele Monda, Maurizio Pieroni, Elena Biagini, Ella Field, Silvia Passantino, Gabriella Dallaglio, Carlo Fumagalli, Panagiotis Antiochos, Ioannis Skalidis, Henri Lu, Ioannis Kachrimanidis, Alessia Argirò, Francesca Girolami, Franco Cecchi, Francesco Cappelli, Perry M Elliott, Juan Pablo Kaski, Giuseppe Limongelli, Iacopo Olivotto

**Affiliations:** Cardiomyopathy Unit, Careggi University Hospital, Largo Brambilla 3, Florence 50121, Italy; Service of Cardiology, University Hospital of Lausanne (CHUV), Lausanne, Switzerland; Inherited and Rare Cardiovascular Diseases Unit, Department of Translational Medical Sciences, University of Campania ‘Luigi Vanvitelli’, Monaldi Hospital, Naples, Italy; Cardiomyopathy Unit, Careggi University Hospital, Largo Brambilla 3, Florence 50121, Italy; Cardiology Unit, IRCCS Azienda Ospedaliero-Universitaria di Bologna, Bologna, Italy; Centre for Paediatric Inherited and Rare Cardiovascular Disease, Institute of Cardiovascular Science, University College London, London, UK; Centre for Inherited Cardiovascular Diseases, Great Ormond Street Hospital, London, UK; Service of Cardiology, Meyer Children’s Hospital IRCCS, Florence, Italy; Cardiomyopathy Unit, Careggi University Hospital, Largo Brambilla 3, Florence 50121, Italy; Cardiomyopathy Unit, Careggi University Hospital, Largo Brambilla 3, Florence 50121, Italy; Service of Cardiology, University Hospital of Lausanne (CHUV), Lausanne, Switzerland; Institut Cardiovasculaire Paris Sud, Mancy, Paris, France; Service of Cardiology, University Hospital of Lausanne (CHUV), Lausanne, Switzerland; Service of Cardiology, Ippokrateio General Hospital, Athens, Greece; Cardiomyopathy Unit, Careggi University Hospital, Largo Brambilla 3, Florence 50121, Italy; Service of Cardiology, Meyer Children’s Hospital IRCCS, Florence, Italy; Cardiomyopathy Unit, Careggi University Hospital, Largo Brambilla 3, Florence 50121, Italy; AICARM Onlus APS, Florence, Italy; Cardiomyopathy Unit, Careggi University Hospital, Largo Brambilla 3, Florence 50121, Italy; Centre for Heart Muscle Disease, Institute of Cardiological Sciences, University College London and St. Bartholomew’s Hospital, London, UK; Centre for Paediatric Inherited and Rare Cardiovascular Disease, Institute of Cardiovascular Science, University College London, London, UK; Centre for Inherited Cardiovascular Diseases, Great Ormond Street Hospital, London, UK; Inherited and Rare Cardiovascular Diseases Unit, Department of Translational Medical Sciences, University of Campania ‘Luigi Vanvitelli’, Monaldi Hospital, Naples, Italy; Cardiomyopathy Unit, Careggi University Hospital, Largo Brambilla 3, Florence 50121, Italy; Service of Cardiology, Meyer Children’s Hospital IRCCS, Florence, Italy

**Keywords:** Hypertrophic cardiomyopathy, Phenocopies, Red flags, App

## Abstract

**Aims:**

Patients with primary left ventricular hypertrophy (LVH) often experience a diagnostic delay of several years, largely related to fragmented knowledge among different specialties and the rarity of the conditions. We developed and validated a digital support tool to guide the physician in the differential diagnostic process of patients presenting with primary LVH.

**Methods and results:**

A total of 818 patients with definitive diagnosis of sarcomeric hypertrophic cardiomyopathy (HCM) or one of its phenocopies [479 (62%) males, 48 ± 24 years] were included. Pre-specified disease-specific red flags (RFs) were categorized into five domains: family history, signs/symptoms, electrocardiography, echocardiographic, and laboratory. Each patient’s characteristics were inserted by two independent and blind investigators into the app. The diagnostic outcome, based on the presence/absence of RF, was categorized as follows: (i) most likely diagnosis, (ii) possible diagnosis, and (iii) less likely diagnosis. A total of 2979 RFs were identified and non-sarcomeric phenocopies exhibited a higher RF burden than sarcomeric HCM (3.9 vs. 2.7 RFs per patient, *P* = 0.007), with systemic features and extracardiac findings being strong predictors of non-sarcomeric disease. Thick-Heart App correctly classified 93% of cases into the most likely diagnosis category (sensitivity of 88–100%, specificity 97%). The positive predictive value (PPV) for TTR amyloidosis reached 92%, while Friedrich’s ataxia was correctly identified in all cases (PPV = 100%).

**Conclusion:**

The Thick-Heart App correctly classified 93% of cases into the most-likely diagnosis category (sensitivity 88–100%, specificity 97%). Our study underscores the potential clinical value of digital decision support tools to enable timelier identification of specific cardiomyopathies, by promoting awareness in non-reference settings.

## Graphical Abstract

**Graphical Abstract:**
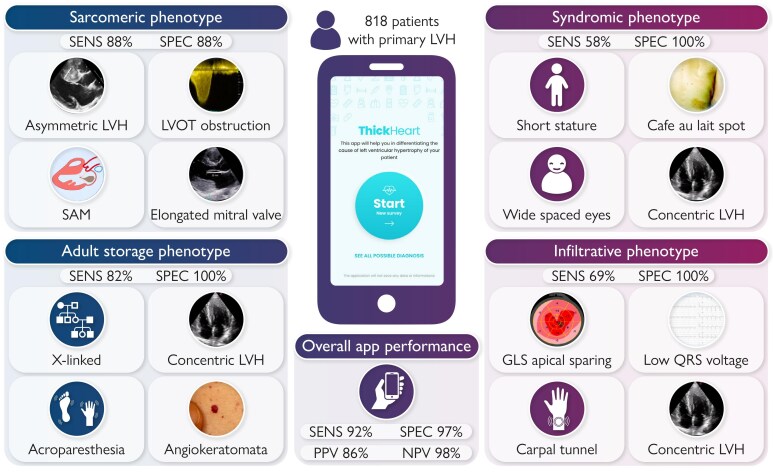

Hypertrophic cardiomyopathy (HCM) is the most common inherited cardiomyopathy and is characterized by a diverse range of phenotypic expression^1,2^ associated with left ventricular hypertrophy (LVH). The aetiologic definition of the cause of the LVH is crucial for the implementation of disease-specific treatments, often with profound impact on symptomatic status and outcome.^3–6^ However, accurate identification of the specific aetiology often represents a challenge, particularly in non-reference centres and in the general cardiology setting. This is mainly related to limited awareness and reduced knowledge of such conditions.^7–10^ For this reason, for patients presenting with LVH, the ESC recommends systematic search for diagnostic clues or ‘red flags’ (RFs) (cardiac and non-cardiac) that can identify particular treatable disorders and guide the appropriate selection of advanced diagnostic tests.^11^ We recently explored and validated this approach in a large cohort of patients presenting with an HCM phenotype.^12^ Despite a universal consensus regarding this approach, however, its potential diagnostic yield in everyday practice remains unclear, mainly because of fragmented knowledge of such conditions.^11,12^ Therefore, in order to try to increase clinical awareness and encourage the diagnostic process, we developed a digital support tool (smartphone-based app) aimed to provide support and guidance to the physician in the differential diagnostic process of patients presenting with primary LVH. Here, we describe the conceptual development of this digital support tool, by determining the predictive performance of RF associated with specific sub-entities in a large cohort of patients with definite diagnoses characterized by a HCM phenotype.^12^

## Methods

### Study population

The present cross-sectional study analysed electrocardiographic, clinical, and echocardiographic data in 818 patients from three referral centres for cardiomyopathies in Europe [Florence (*n* = 479), Naples (*n* = 224), and London (*n* = 115)] from 2015 to 2023, as previously described.^12^ For the specific purpose of this study, only patients with definite diagnoses of sarcomeric HCM or one of its phenocopies, according to standardized protocols comprising non-invasive/invasive investigations including tissue biopsy (if necessary) and genetic testing, were included. The study was approved by the institutional IRB, and informed consent was obtained by patients.

### Inclusion criteria

Diagnosis of HCM was based on two-dimensional echocardiographic evidence of a hypertrophied, non-dilated LV (maximum wall thickness ≥15 mm, or Z score > 2 SD), in the absence of another cardiac or systemic disease capable of producing the magnitude of hypertrophy evident.^13^ All patients underwent genetic testing for sarcomeric HCM and its most common phenocopies associated. Those presenting with a sarcomeric HCM phenotype but without pathogenic/likely pathogenic disease-causing variant were included and called ‘genotype elusive HCM’.^13^ Syndromic, metabolic, inﬁltrative, and neuromuscular disorders associated with HCM were deﬁned as ‘non-sarcomeric causes of HCM’. This group included HCM phenocopies defined according to the ESC deﬁnition.^13^ Patients with uncontrolled hypertension were excluded. A panel of diagnostic markers was deﬁned using recommendations from the ESC position statement on diagnosis of cardiomyopathies and the ESC HCM and cardiomyopathy guidelines.^11–13^ Individual markers, or diagnostic RF were systematically assessed and organized into ﬁve groups: family history, signs and symptoms, electrocardiography, echocardiography, and laboratory testing.^12^

*Relevant RFs* were defined as clinical or instrumental characteristics with a specificity of >75% for the associated disease, as previously described.^12^

*Normal RFs* were categorized as those being associated with the specific HCM aetiology, but with a specificity of <75%. A total of 5902/63 804 (9.2%) data points were missing.

### Application development and algorithm description

The smartphone-based app was conceptualized as a digital support and didactic tool to guide the physician in the differential diagnostic process of patients presenting with primary LVH. The app—named ‘Thick-Heart’—after recalling the exclusion of most common secondary causes of LVH, guides the user through five domains of RF: family history, signs/symptoms, ECG, echocardiography, and laboratory. Each RF is defined *a priori* based on specificity (>75% for ‘relevant’ RF; <75% for ‘normal’ RF) from prior published studies.^11–13^ Once the clinician inputs yes/no for each RF, the app applies a deterministic algorithm to assign every target aetiology [sarcomeric HCM, TTR amyloidosis, Fabry disease (FD), etc.] to one of the three categories:

1. Most likely diagnosis: Patient’s age is within the predefined age range for that condition and at least one ‘relevant’ RF is marked or more than one ‘normal’ RF is marked.
2. Possible diagnosis: Patient’s age is within the predefined age range and at least one ‘normal’ RF is marked (but no ‘relevant’ RF).
3. Less likely diagnosis: Patient’s age is outside the predefined age range and no RF is marked.

**Figure 1:**
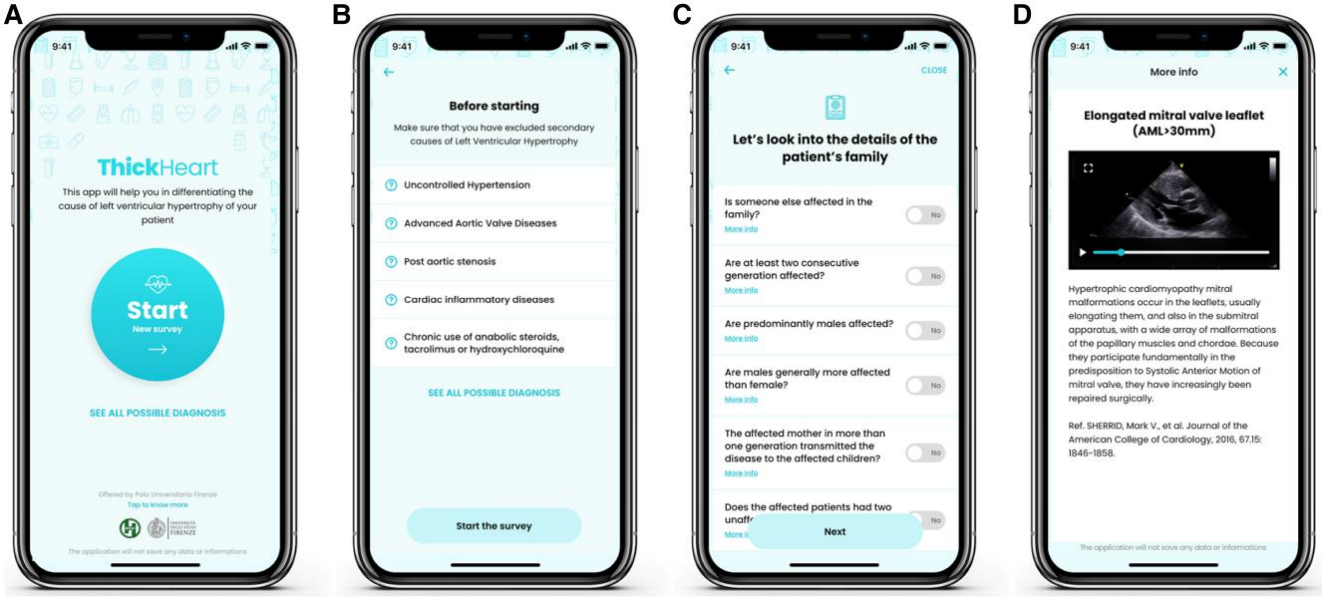
Example of ‘Thick-Heart’ App user interfaces and functioning. (*A*) The opening screen is represented. (*B*) The second screen describing the exclusion of the secondary causes of left ventricular hypertrophy. (*C*) The interface of the inquiry about the anamnesis and family history of the examined patients. (*D*) Example of a multimedia content, in this case ‘Elongated mitral valve leaflet (AML > 30 mm) available in the app. HCM, hypertrophic cardiomyopathy; TTR, transthyretin; LVH, left ventricular hypertrophy; GLS, global longitudinal strain.

The algorithm uses a rule-based approach (i.e. each RF is treated as a binary input and combined via Boolean logic), which differs from Bayesian or other probabilistic models, since there is no intermediate ‘probability score’ or weighted updating. Instead, the app mimics the stepwise clinical reasoning recommended by the ESC guidelines,^11–13^ which shall mimic the ‘cardiomyopathy mindset’ approach (*Figure 1*). All age boundaries and specificity results for each RF are provided in Supplementary material online, *Table S1*. The ultimate objective of the present algorithm is a free diffusion by partnering with patient advocacy or non-profit organization to ensure free access for clinicians, after that regulatory approval (Class IIA medical device following European MDR Regulation) will be granted.

### Algorithm development and testing

Two independent users (N.M. and P.A.) inserted each patient data and RF in the ‘Thick-Heart’ App, blinded to aetiology of HCM and reporting possible crashes or bugs of the app. Outcomes of the app algorithm were recorded and then compared with the actual specific diagnosis of the patient. Correct identification of the specific aetiology for the patient was accepted if the app algorithm classified the patients in the *most likely diagnosis group* (true positive). Operational usability was assessed by three different certified cardiologists (H.L., I.S., and I.K.).

### Statistical analyses

The clinical significance of each RF was calculated within the relevant clinical condition. Due to the stringent selection criteria, patients were not consecutively enrolled; the prevalence of HCM phenocopies was intentionally over-represented, compared with unselected populations, to include a meaningful proportion of rare diseases. Continuous variables, reported as means with standard deviations or as medians with interquartile ranges for non-normal distributions, were compared between groups with Student’s *t*-test or non-parametric tests, as appropriate. Categorical variables, reported as counts and percentages, were compared between groups with *χ*² or Fisher exact tests.

Sensitivity (Se), speciﬁcity (Sp), positive predictive value (PPV), and negative predictive value (NPV) of RF for speciﬁc causes of HCM were analysed. In brief, (i) for all RF, we calculated the clinical significance of any single RF to detect the presence of any speciﬁc (non sarcomeric) aetiology of HCM (i.e. Se, Sp, PPV, NPV); (ii) for single RF and clusters, we calculated the Se, Sp, PPV, and NPV of RF to detect the speciﬁc aetiology. To estimate a single ‘overall’ performance metric for the app—given that it generates separate sensitivity, specificity, PPV, and NPV values for each individual diagnosis—we computed prevalence-weighted averages across all target conditions in our 818-patient cohort.

A two-sided *P* < 0.05 was considered statistically significant. All analyses were performed using SPSS Statistics for Macintosh version 25.0 (IBM).

## Results

### Age, clinical, and demographic profile of the cohort

The study cohort comprised a total of 818 [479 (58%) males] patients with a HCM phenotype, with a mean age at diagnosis of 49 ± 21 years. The different aetiologies were represented as follows: 424 (52%, 46 ± 18 years, range 0–79 years, of whom 314 genetically proven) with sarcomeric HCM; 139 (20%, 76 ± 7 years, range 51 to 88 years) with TTR amyloidosis; 101 (14%, 44 ± 11 years, range 22 to 68 years) with FD; 34 (5%, 12 ± 7 years, range 0–34 years) with Noonan syndrome; 30 (4%, 18 ± 7 years, range 14–29 years) with Friedreich’s ataxia; 21 (3%, 6 ± 4 years, range 0–14 years) with mitochondrial disease; 19 (3%, 16 ± 10 years, range 0–31 years) with Danon disease; 18 (3%, 3 ± 2 years, range 0–9 years) with Noonan syndrome with multiple lentigines (NSML); 16 (2%, 2 ± 1 years, range 0–5 years) with Pompe disease; and 16 (1%, 16 ± 8 years, range 2–34 years) with *PRKAG2* cardiomyopathy (*Table 1*). Patients with TTR cardiomyopathy showed a more dilated left atrium (mean anteroposterior diameter 47 ± 12 mm, vs. 40 ± 22 mm for the rest of the cohort, *P* < 0.01) and lower ejection fraction (58 ± 7% vs. 64 ± 6%, *P* < 0.01). Danon disease patients presented the highest maximal left ventricular (LV) wall thickness (25 ± 8 mm, compared with 20 ± 5 mm for the rest of the cohort, *P* < 0.01) (*Table 1*). A total of 109 (13%) patients had paediatric-onset HCM, with LV maximal wall thickness Z score of 12 (IQR 9; 18), relative to a reference population of the same age, body mass index, and body surface area.^13^

**Table 1:** Clinical and demographic characteristics of patients included in the cohort

	Variable	Total cohort (*n* = 818)	Sarcomeric HCM (*n* = 318)	Genotype elusive HCM (*n* = 106)	Anderson–Fabry disease (*n* = 101)	TTR amyloidosis (*n* = 139)	Danon (*n* = 19)	Friederich’s ataxia (*n* = 30)	Noonan syndrome with multiple lentigines (*n* = 18)	Mitochondriopathies (*n* = 21)	Pompe (*n* = 16)	Noonan (*n* = 34)	PRKAG2 (*n* = 16)
Clinical	Male gender	477 (58%)	175 (55%)	65 (61%)	64 (63%)	86 (62%)	11 (60%)	20 (67%)	8 (46%)	14 (67%)	7 (43%)	23 (67%)	6 (39%)
	Age at diagnosis (years)	49 ± 21	43 ± 16	48 ± 17	44 ± 11	76 ± 7	16 ± 10	18 ± 7	3 ± 2	6 ± 4	2 ± 1	12 ± 7	16 ± 8
	NYHA I (*n*)	374 (46%)	158 (49%)	49 (46%)	68 (68%)	32 (23%)	7 (37%)	11 (37%)	8 (44%)	10 (48%)	5 (31%)	17 (50%)	11 (69%)
	NYHA II (*n*)	312 (38%)	130 (40%)	38 (36%)	23 (23%)	59 (42%)	5 (26%)	15 (50%)	7 (39%)	10 (48%)	8 (50%)	14 (41%)	5 (31%)
	NYHA III (*n*)	121 (15%)	38 (12%)	12 (11%)	10 (9%)	44 (32%)	6 (32%)	3 (10%)	3 (17%)	1 (4%)	3 (39%)	3 (9%)	0
	NYHA IV (*n*)	9 (1%)	2 (1%)	7 (7%)	0	5 (3%)	1 (5%)	1 (3%)	0	0	0	0	0
	Hypertension (*n*)	295 (36%)	135 (42%)	45 (42%)	35 (35%)	77 (55%)	0	1 (3%)	0	1 (4%)	0	0	1 (6%)
	Diabetes (*n*)	42 (5%)	16 (5%)	5 (5%)	5 (5%)	15 (11%)	0	0	0	1 (4%)	0	0	0
	eGFR^a^ (mL/kg/min/m^2^)	63 ± 18	67 ± 11	67 ± 16	56 ± 25	75 ± 32	43 ± 10	45 ± 17	33 ± 21	43 ± 12	42 ± 15	41 ± 17	44 ± 18
	Negative family history	337 (41%)	178 (43%)	35 (32%)	23 (23%)	68 (45%)	6 (60%)	13 (42%)	8 (46%)	10 (48%)	8 (50%)	17 (50%)	8 (50%)
	Autosomal dominant	347 (51%)	246 (71%)	29 (27%)	0	76 (55%)	0	0	10 (54%)	0	0	17 (50%)	8 (50%)
	Autosomal recessive	25 (4%)	0	0	0	0	0	17 (58%)	0	0	8 (50%)	0	0
	X-linked	90 (12%)	0	0	77 (77%)	0	13 (70%)	0	0	0	0	0	0
	Matrilinear	17 (1%)	0	0	0	0	0	0	0	17 (81%)	0	0	0
Echocardiographic	Left atrium diameter (mm)	41 ± 24	42 ± 13	45 ± 14	44 ± 11	47 ± 12	39 ± 10	34 ± 17	31 ± 11	38 ± 14	25 ± 12	28 ± 7	36 ± 8
	Maximal LV wall thickness (mm)^b^	20 ± 6	21 ± 9	21 ± 11	18 ± 5	18 ± 4	25 ± 8	16 ± 3	19 ± 10	18 ± 6	20 ± 8	18 ± 5	18 ± 4
	Ejection fraction (%)	64 ± 6	66 ± 8	65 ± 8	63 ± 6	58 ± 7	68 ± 10	62 ± 7	63 ± 5	59 ± 4	62 ± 6	61 ± 7	58 ± 8

HCM, hypertrophic cardiomyopathy; TTR, transthyretin; eGFR, estimated glomerular filtration rate; LV, left ventricle.

^a^eGRF was available for 402/818 (49%) of patients.

^b^For paediatric patients, Z scores are reported in the text.

### Prevalence of disease-specific red flags

**Figure 2:**
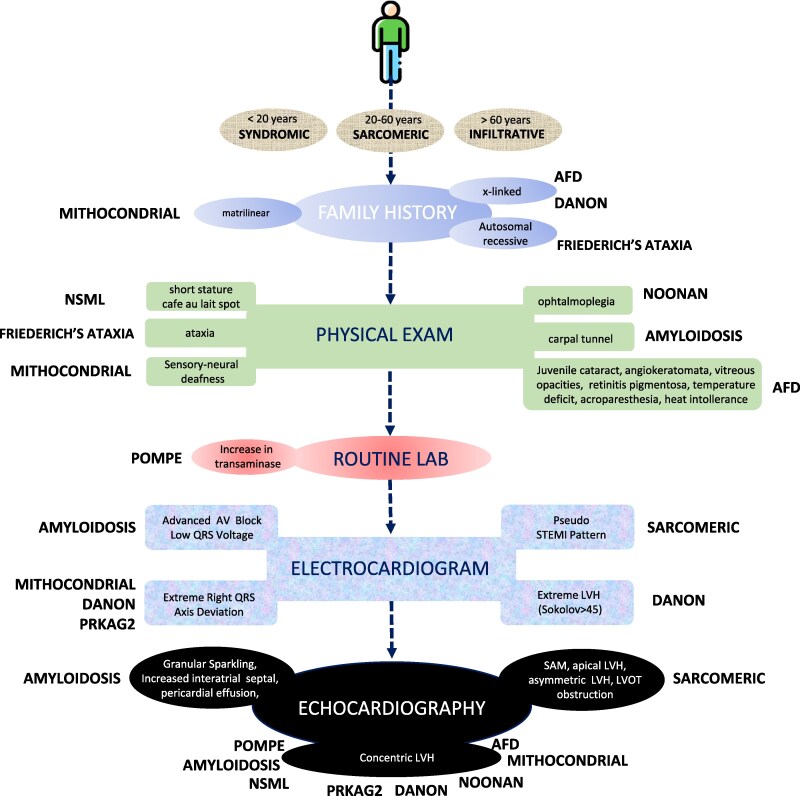
Summary of relevant red flags (specificity > 75%) triggering diagnostic suspicion of specific cause of hypertrophic cardiomyopathy. SAM, systolic anterior motion of the mitral valve; LVH, left ventricular hypertrophy; LVOT, left ventricular outflow.

Overall, systematic analysis of signs and symptoms led to the identification of a total of 2979 RFs, an average of three per patient. Patients with sarcomeric HCM had a median of 2.7 RF per person whereas patients with non-sarcomeric aetiologies had a mean of 3.7 RF (*P* = 0.07). *Relevant RFs* (those with a specificity > 75%) identified are summarized in *Figure 2*.

#### Family history and age

In total, 1018/2979 (34%) RFs were identified by clinical history, systematic physical examination, and routine laboratory tests. X-linked transmission was almost exclusively observed in patients with FD and Danon patients [77 (77%), Sp 81% and 8 (40%), Sp 86%, respectively]. Matrilinear transmission was found only in patients with mitochondrial disease (Sp 82%), whereas autosomal recessive inheritance a high specificity was demonstrated for patients with Friedreich’s ataxia (17/30, 58%, Sp 92%) (see Supplementary material online, *Table S1A*).

Age at diagnosis was important in distinguishing different aetiologies. A diagnosis in patients aged <20 years was specific for all the syndromic causes of HCM (Danon, Friederich’s ataxia, NSML, mitochondriopathies, Pompe, Noonan, and PRKAG2) (see Supplementary material online, *Table S1A*). Sarcomeric HCM and genotype elusive HCM were diagnosed most commonly in middle aged patients (Sp 78% and 75%, respectively). A diagnosis of HCM after age 60 carried a Sp of 86% for TTR cardiomyopathy (see Supplementary material online, *Table S1A*).

#### Systematic physical examination and routine laboratory analysis

Three-hundred and fifty (11%) RFs were identified during systematic physical examination and 166 (6%) with routine laboratory tests.

*Relevant RFs* were identified for FD, including cornea verticillate, identified in 19/101 (18%, Sp 99%) FD patients. In addition, retinitis pigmentosa was found in 14/16 (88%, Sp 99%) patients with Danon disease. Temperature sensitivity deficit and acroparesthesia were found exclusively in FD patients (Sp 100%) (see Supplementary material online, *Table S1A* and *B*), whereas gait disturbances identified only in patients with Friedreich’s ataxia (30/30). Biceps tendon rupture present only in TTR cardiomyopathy [12/139 (8%), Sp 100%]. Finally, routine laboratory abnormalities suggesting specific aetiologies were found in 189/818 (23%) patients. Of note, raised transaminase levels were found in 11/16 (69%, Sp 99%) of patients with Pompe disease (see Supplementary material online, *Table S1B–D*; *Figure 2*).

#### Electrocardiogram and echocardiogram analysis

A total of 491 ECG RFs were identified (16% of all RF). Advanced conduction abnormalities were present in 72 (9%) patients, and it was a *relevant RF* for TTR cardiomyopathy (Sp 79%) (see Supplementary material online, *Table S1B*). Extreme right axis deviation was rare (18 cases, 2%) and extremely specific for Danon disease (Sp 93%), *PRKAG2* cardiomyopathy (Sp 92%), metabolic disease (Sp 90%), and Noonan syndrome (Sp 85%). Low QRS voltages were present in 40 (5%) patients, and extremely specific for TTR cardiomyopathy (Sp 95%) (see Supplementary material online, *Table S1C*; *Figure 2*).

A total of 1470 RFs (49% of all RF) were identified by standard echocardiography. Mitral valve SAM was highly specific for sarcomeric HCM (Sp 80%). Five (0.6%) patients presented with mid-ventricular obstruction, all with sarcomeric HCM (see Supplementary material online, *Table S1C*; *Figure 2*).

The pattern of hypertrophy was important in discriminating the different aetiologies: while isolated apical hypertrophic was only observed in sarcomeric and genotype elusive HCM (48/48 patients), concentric LVH and severe left posterior wall thickness were observed almost exclusively in non-sarcomeric HCM [332/344 (97%) and 39/39 patients, respectively]. Asymmetric LVH was specific for the identification of sarcomeric and genotype elusive HCM (Sp 80% and 79%, respectively) (see Supplementary material online, *Table S1C*; *Figure 2*).

### Diagnostic performance of the ‘Thick-Heart’ App

*Relevant RFs* for each disease, i.e. those with a specificity of more than 75%, are summarized in *Figure 2*. The clinical values of RF for the five main clusters of HCM subtypes are shown in *Table 2*.

**Table 2:** Sensitivity, specificity, and positive and negative predictive value of clusters of specific red flags triggering disease-specific diagnostic process

	Cluster of red flags (specificity > 75%)	Sensitivity	Specificity	Positive predictive value	Negative predictive value
Sarcomeric phenotype (genotype + HCM and genotype elusive HCM)	‘Pseudo-STEMI’ pattern, SAM, elongated anterior mitral leaflet, apical LVH, asymmetric LVH, LVOT obstruction	86% [95% CI 83–94%]	99% [95% CI 93–100%]	99% [95% CI 96.4–100%]	94% [95% CI 91–98%]
Adult storage phenotype (Anderson–Fabry disease)	Age, X-linked inheritance, juvenile cataract, angiokeratomata, vitreous opacities, retinitis pigmentosa, T°-sensitive deficit, juvenile stroke, acroparesthesia, heat intolerance, concentric LVH	82% [95% CI 79–88%]	100% [95% CI 98–100%]	100% [95% CI 96–100%]	86% [95% CI 84–89%]
Infiltrative phenotype (TTR amyloidosis)	Age, carpal tunnel, advanced AV block, low QRS voltage, concentric LVH, increased interatrial septum thickness, granular sparkling, pericardial effusion	69% [95% CI 62–76%]	100% [95% CI 96–100%]	100% [95% CI 97–100%]	98% [95% CI 94–100%]
Paediatric storage/lysosomal phenotype (Pompe, Danon)	Age, X-linked, extreme right QRS axis deviation, concentric LVH, extreme LVH (Sokolov > 45), increase in transaminase	75% [95% CI 79–88%]	100% [95% CI 98–100%]	100% [95% CI 96–100%]	88% [95% CI 85–92%]
Syndromic phenotype (Friederich’s ataxia, Noonan syndrome with multiple lentigines, mitochondrial diseases, Noonan)	Age, autosomal recessive and matrilinear inheritance, ophthalmoplegia, ocular hypertelorism, growth retardation, lentigines/cafe au lait spot, ataxia, rapid progression of LVH, concentric LVH, increase in transaminase	58% [95% CI 51–63%]	100% [95% CI 98–100%]	100% [95% CI 99–100%]	91% [95% CI 89–96%]

For sensitivity, specificity, and positive and negative predictive value, 95% confidence intervals are provided.

HCM, hypertrophic cardiomyopathy; TTR, transthyretin; SAM, systolic anterior motion of the mitral valve; LVH, left ventricular hypertrophy; LVOT, left ventricular obstruction; AV, AV block; T°, temperature.

Seventeen (1%) bugs (app crashes) occurred during this process. A total of 760/818 (93%) were correctly identified by the app algorithm and categorized in the group ‘Most likely diagnosis’ (*Table 3*). Sensitivity was very high for each specific aetiology, ranging from 88% for TTR cardiomyopathy to 100% for Friederich’s ataxia. Overall specificity was 97% (*Table 3* and *Graphical Abstract*).

**Table 3:** Outcome and specificity, sensibility, and positive and negative predictive values of the ‘Thick-Heart App’ algorithm in the identification of specific causes of hypertrophic cardiomyopathy

Algorithm performance	Sarcomeric HCM (*n* = 318)	Genotype elusive HCM (*n* = 106)	Anderson–Fabry disease (*n* = 101)	TTR amyloidosis (*n* = 139)	Danon (*n* = 19)	Friederich’s ataxia (*n* = 30)	Noonan syndrome with multiple lentigines (*n* = 18)	Mitochondriopathies (*n* = 21)	Pompe (*n* = 16)	Noonan (*n* = 34)	PRKAG2 (*n* = 16)
Category—most likely diagnosis	306	101	93	122	17	30	16	18	15	29	13
Category—possible diagnosis	355	596	539	494	412	289	301	329	376	334	401
Category—least likely diagnosis	157	121	276	102	389	499	501	471	426	455	404
True positive	306	101	93	122	17	30	16	18	15	29	13
False positive	38	19	22	18	3	2	1	4	3	2	10
True negative	488	564	457	432	674	690	635	651	699	654	598
False negative	12	5	8	17	2	0	2	3	1	5	3
Sensibility	0.96	0.95	0.92	0.88	0.89	1	0.89	0.86	0.94	0.85	0.81
Specificity	0.93	0.96	0.95	0.96	0.99	0.99	0.99	0.99	0.98	0.99	0.97
Positive predictive value	0.89	0.84	0.81	0.87	0.85	0.94	0.94	0.82	0.83	0.94	0.60
Negative predictive values	0.98	0.99	0.98	0.96	0.99	1	0.99	0.99	0.99	0.99	0.99

HCM, hypertrophic cardiomyopathy; TTR, transthyretin.

## Discussion

**Figure 3:**
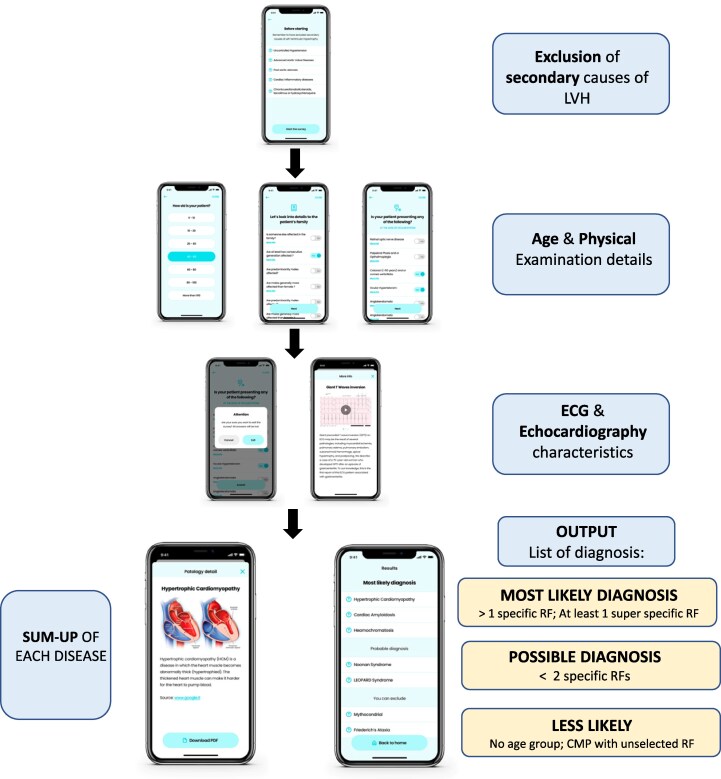
Simplified flowchart of ‘Thick-Heart’ app workflow with its users interface. LVH, left ventricular hypertrophy; ECG, electrocardiogram; RF, red flag.

Elucidation of a specific cause for a cardiomyopathy can directly influence management of patients and their relatives and is the basic tenet of the approach to diagnosis and management recommended in the 2023 ESC Cardiomyopathy Guidelines.^13^ Outside non-reference settings, once a morphological diagnosis has been made, further investigations are often protocol rather than hypothesis driven, but this approach can fail to identify an underlying disease mechanism. For this reason, we designed a digital tool aimed at supporting the physician in a hypothesis-driven diagnostic approach of patients presenting with primary LVH. The rationale lies in the fact that deliberate analysis of every aspect of the individual and their family as well as an integrated probabilistic interpretation of cardiac investigations is required to guide the diagnostic process (*Figure 3*). This approach, proposed by the ESC,^11^ includes a systematic search for diagnostic clues or RF (cardiac and non-cardiac) that can identify particular treatable disorders. We recently validated this strategy in a large cohort of patients presenting with an HCM phenotype^12^ and highlighted that over than 34% of individual RF (mostly relevant to rare HCM phenocopies) were potentially identifiable in a non-tertiary setting by careful anamnesis, physical examination, and family history. In such an environment, gaps in knowledge can be present and in many cases might represent the failure to the application of this scheme. Therefore, by harnessing the power and diffusion of smartphone technology, we designed an app for the cultural support of physicians who are not confronted on a daily based with patients presenting with LVH. The app—named ‘Thick-Heart’—after recalling the exclusion of most common secondary causes of LVH, guides the user through the anamnesis, family history, ECG, echocardiographic characteristics, and laboratory results commonly associated with HCM and its phenocopies (*Figure 1*). The user can record and insert whether a given RF is present in the patient being analysed. For each RF, a specific pop-up can be opened, with an explanation with bibliographic reference and multimedia content related to it (*Figure 1*). Once the anonymized patient’s RF are inserted, the app algorithm suggests three possible scenarios, based on the RF inserted, describing the most likely, probable, and less likely diagnosis.

### Clinical significance of different red flags

Red flags were a common finding, with an average of 3.5 RF, more often seen in patients with non-sarcomeric compared with sarcomeric HCM (mean 3.7 vs. 2.7, *P* = 0.07). However, not all RFs carried the same diagnostic weight, and their significance varies depending on the context in which they appear, their specificity for a particular disease, and their ability to direct targeted investigations. Specifically, the identification of RF during physical examination was strongly associated with a non-sarcomeric aetiology, whereas most (68%) RFs associated with sarcomeric HCM were derived from ECG and echocardiography. Moreover, we found that a significant sub-group of RF was extremely specific for a given specific aetiology, with a specificity > 75%. Age at diagnosis identified three different diagnostic clusters, as patients with syndromic causes of HCM were diagnosed almost exclusively under the age of 20, whereas those with infiltrative/storage phenotypes presented after the age of 60 (except for Pompe disease, diagnosed in infancy), in line with previous reports.^8,14,15^ Specific patterns of inheritance were observed, and matrilinear patterns, autosomal recessive, and X-linked transmission were strongly associated with mitochondrial disease, Friedreich’s ataxia, and AFD, respectively. Physical examination was important in AFD, since it was associated with specific facial, neurological, and cutaneous signs. Overall, syndromic causes of HCM presented relevant non-cardiac signs, such as neurological involvement in mitochondrial disease and Friedreich’s ataxia or cutaneous signs in NSML. In contrast, ECG abnormalities were common and useful for the diagnostic suspicion of specific aetiologies. Advanced conduction abnormalities were associated with TTR-CA, and extreme right axis deviation was rare in our cohort (2%) but described only in patients with Danon disease and PRKAG2 cardiomyopathy (*Figure 2*). Standard echocardiography proved very helpful in the identification of RF associated with sarcomeric HCM, such as mitral valve disease (SAM and anterior mitral leaflet elongation). The distribution of hypertrophy played a crucial role in distinguishing between different aetiologies. Isolated apical hypertrophy was found exclusively in cases of sarcomeric HCM, whereas concentric LVH and marked thickening of the left posterior wall were observed predominantly in non-sarcomeric forms of HCM (*Figure 2*). Our findings suggest that a significant proportion of non-sarcomeric HCM aetiologies can be identified with a high degree of specificity through a detailed patient history and physical examination, even in a non-reference setting. This challenges the prevailing assumption that distinguishing between HCM subtypes requires advanced diagnostic modalities. While cutting-edge imaging techniques and genetic testing remain valuable for confirming a diagnosis, their primary role is often validating an already well-founded clinical suspicion. Optimizing diagnostic resource utilization is essential, as early recognition of RF can guide timely referrals to specialized centres, improving the likelihood of prompt diagnosis and targeted therapeutic interventions.^16^

### Diagnostic performance of the application and its intended use

The ‘Thick-Heart App’ demonstrated high diagnostic performance, correctly classifying 93% of cases into the most likely diagnosis category. Sensitivity varied depending on the specific aetiology of LVH, showing particularly high values for infiltrative and metabolic disorders such as TTR cardiomyopathy (88%) and Friedrich’s ataxia (100%), while maintaining an overall specificity of 97% (*Table 3*). These findings suggest that the structured, RF-based algorithm employed by the app effectively aids in the systematic exclusion of unlikely diagnoses, while refining differential possibilities based on the most informative clinical features. Importantly, the stepwise, logic-driven decision-making framework embedded in the app mimics the reasoning process of experienced cardiologists of reference centres, ensuring that RFs with the highest predictive value are prioritized, thereby reducing diagnostic uncertainty.

The intended use of the present digital support tool is expected to reduce variability in clinical practice by providing a standardized framework for differential diagnosis, particularly in cases where multiple RFs are present. Given that HCM and its phenocopies often require reference centres expertise to differentiate, a decision support tool capable of providing physician with a structured and systematic approach through the search for RF can be important for cardiologist outside referral centres for cardiomyopathies. Such strategy aims at eliciting the interest for specialized non-cardiac features (e.g. cornea verticillate, vitreous opacities, carpal tunnel syndrome, juvenile strokes, hypohidrosis) by targeted history, a more extensive physical examination (presence of café au lait spots or growth retardation and short stature), a critical read of the ECG and echocardiogram (short PR interval, extreme axis deviations, low QRS voltages or specific strain patterns).

Real-world implementation of the Thick-Heart App can be a valuable addition to ‘fill’ the inevitable existing gap in knowledge between expert centres and community practice, where probably most of patients are seen for the first time. Lastly, such smartphone-based tool could potentially support the implement the guidelines^13^ in clinical practice by prioritizing the second-line investigation only to those in most need of in low to middle income settings, where the access is often limited.^17^

### Limitations

Several limitations should be acknowledged. First, the study cohort consisted of patients with definitive diagnoses of HCM or its phenocopies, leading to an overrepresentation of rare conditions compared with the general LVH population. As a result, our findings may overestimate the prevalence of RFs in real-world clinical practice. Second, the app needs an external validation from another cohort of patients presenting with an HCM phenotype, to understand its external applicability also in non-white ethnicity. Third, while the app’s diagnostic performance was initially assessed, its impact on actual clinical decision-making and patient management was not assessed. Future prospective studies are needed to determine whether RF-based digital screening improves diagnostic efficiency and leads to tangible patient benefits.

## Conclusions

In conclusion, RFs are a common finding in patients presenting with an HCM phenotype, with a high degree of specificity for distinguishing sarcomeric from non-sarcomeric aetiologies. The Thick-Heart App correctly classified 93% of cases into the most likely diagnosis category, with a sensitivity ranging from 88 to 100% and an overall specificity of 97%. By leveraging smartphone technology and structured diagnostic algorithms, our study underscores the potential clinical value of digital decision support tools to enable timelier identification of specific cardiomyopathies, by promoting awareness in non-reference settings.

## Supplementary Material

ztaf105_Supplementary_Data

## Data Availability

Data would not be available.
