# Supplementary material for: Development of a smartphone-based app to support the differential diagnosis in patients with primary left ventricular hypertrophy
Source: Eur Heart J Digit Health. 2025 Sep 16;7(1):ztaf105. doi: 10.1093/ehjdh/ztaf105 (PMC12821060; doi:10.1093/ehjdh/ztaf105)

**SUPPLEMENTARY MATERIALS**

**Tab. 1 A Supplementary** Sensibility and Specificity of predefined **r**elevant Red Flags for Sarcomeric and non-sarcomeric HCM triggering specific diagnostic suspicion

*Abbreviations: HCM: Hypertrophic Cardiomyopathy; TTR: Transthyretin; LVH: Left ventricular Hypertrophy.*

**
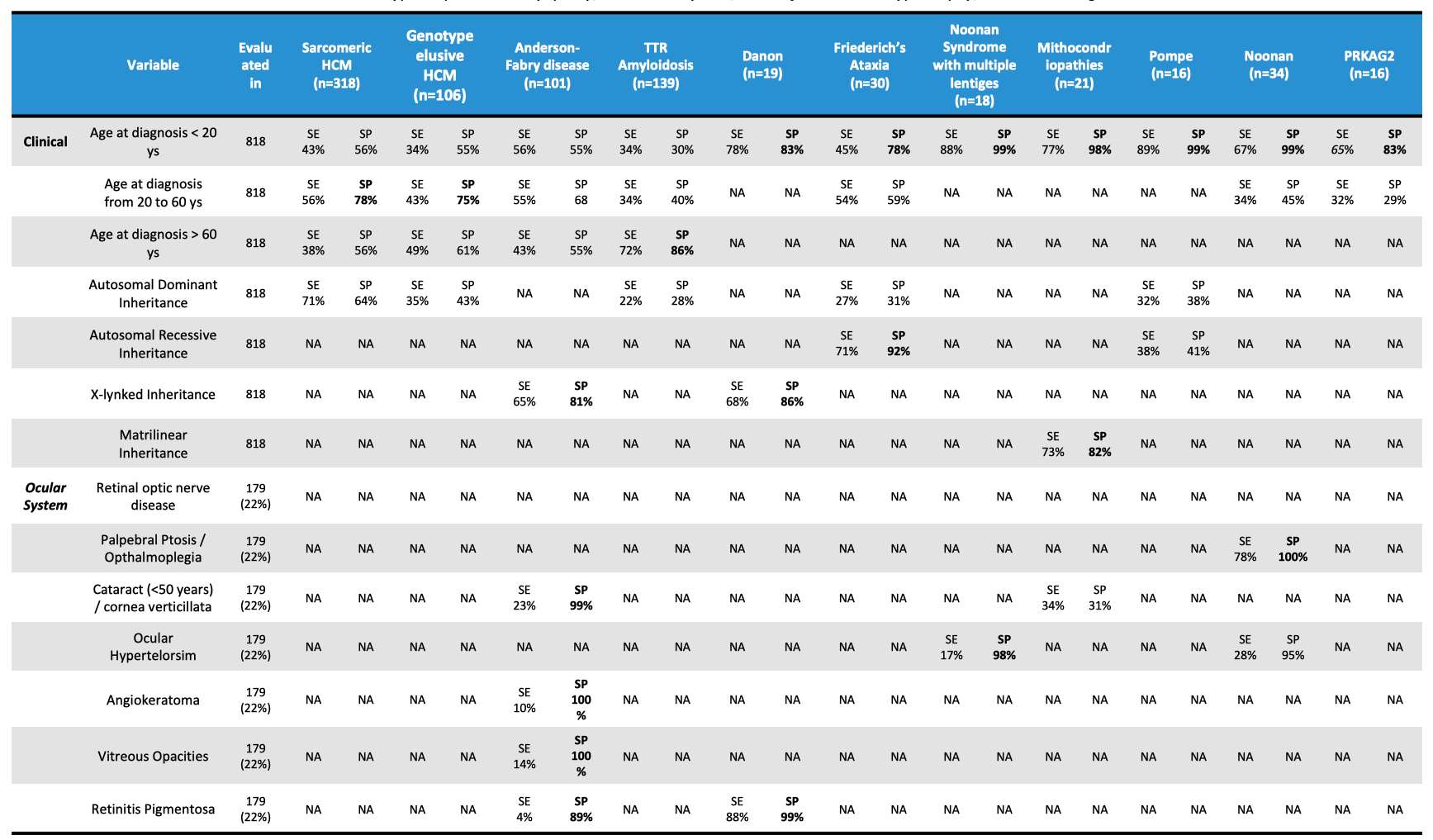
**

**Tab. 1 B Supplementary** Sensibility and Specificity of predefined **r**elevant Red Flags for Sarcomeric and non-sarcomeric HCM triggering specific diagnostic suspicion

*Abbreviations: HCM: Hypertrophic Cardiomyopathy; TTR: Transthyretin; LVH: Left ventricular Hypertrophy.*


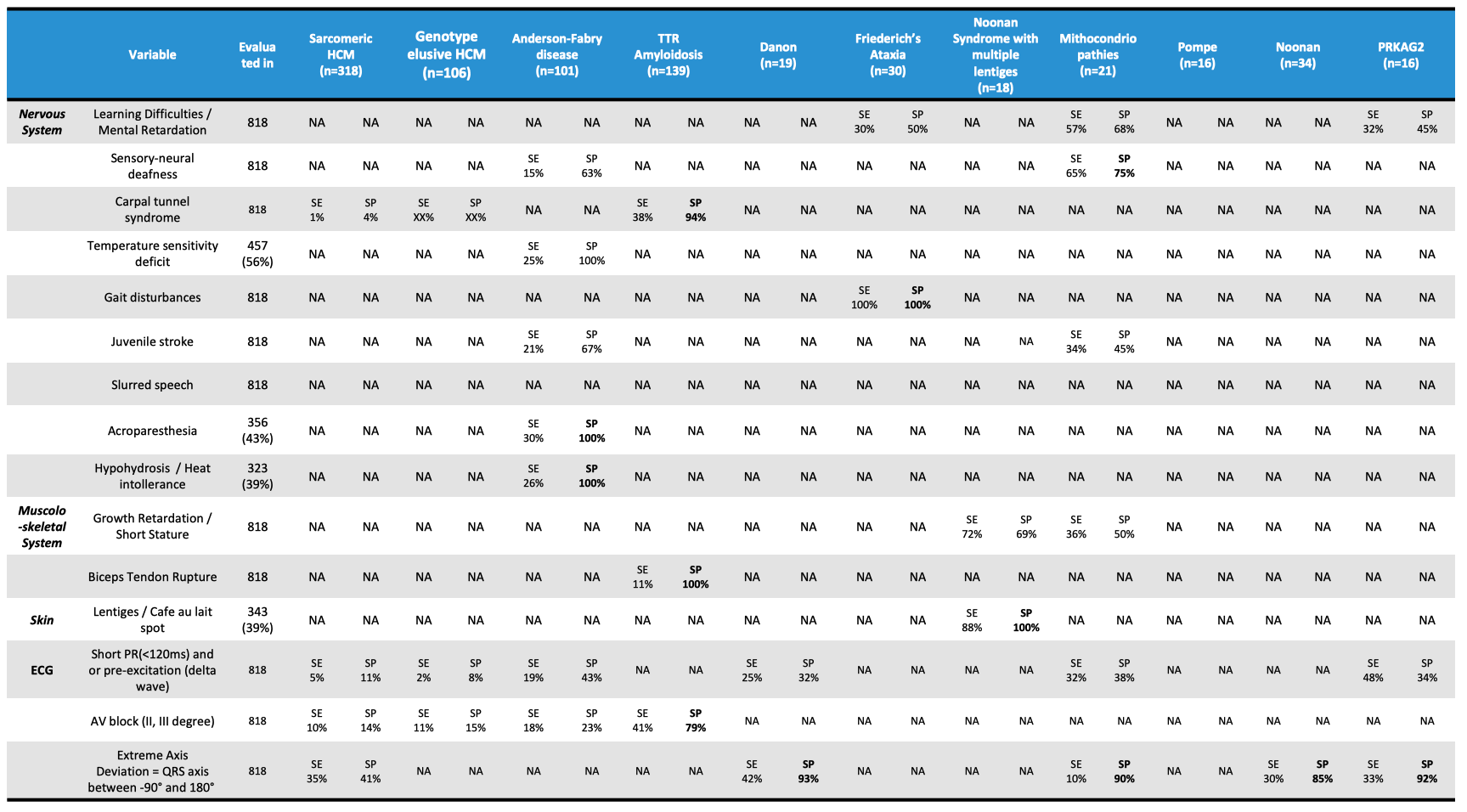


**Tab. 1 C Supplementary** Sensibility and Specificity of predefined **r**elevant Red Flags for Sarcomeric and non-sarcomeric HCM triggering specific diagnostic suspicion

*Abbreviations: HCM: Hypertrophic Cardiomyopathy; TTR: Transthyretin; LVH: Left ventricular Hypertrophy; GLS: Global longitudinal strain.*


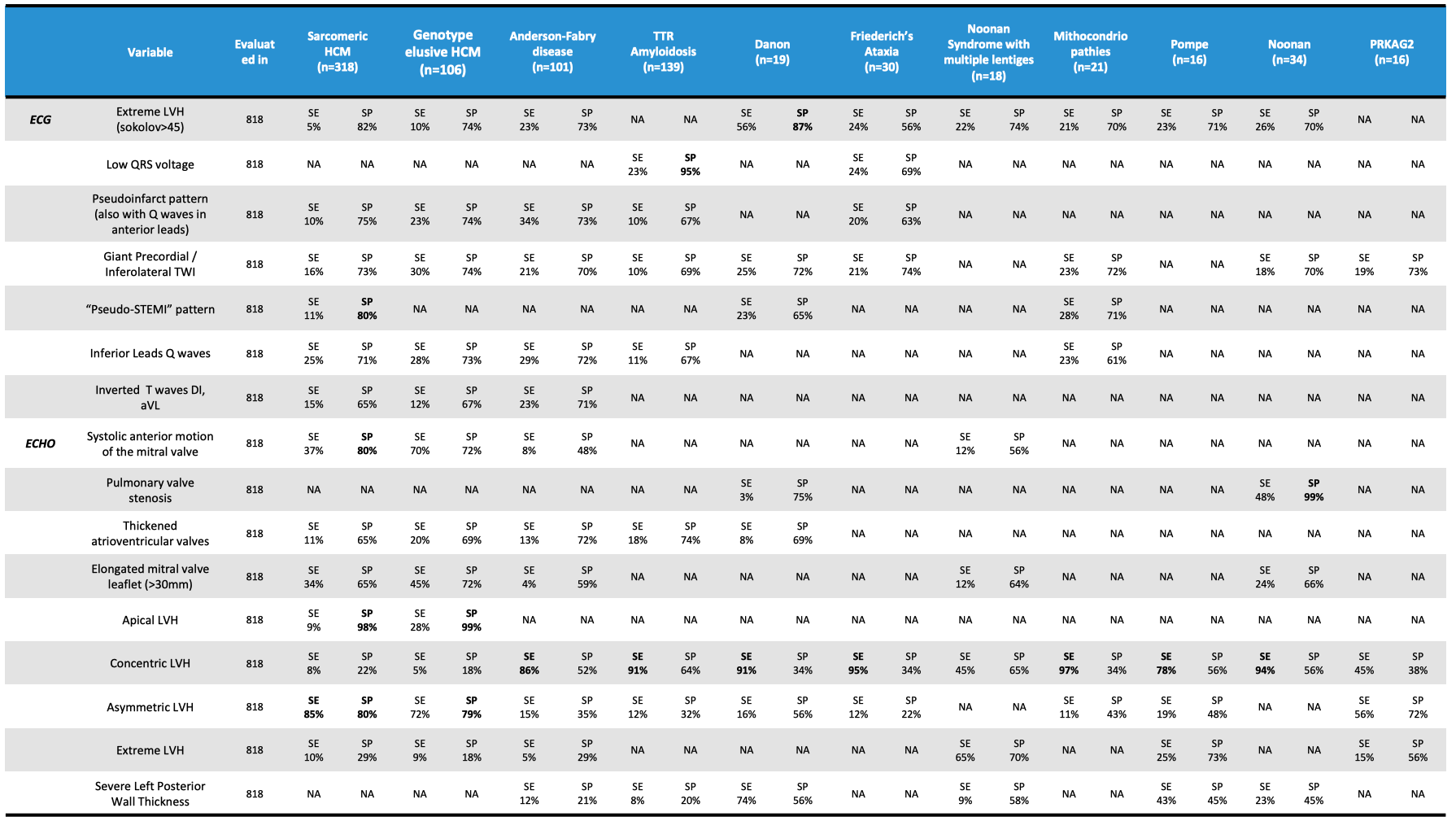


**Tab. 1 D Supplementary** Sensibility and Specificity of predefined **r**elevant Red Flags for Sarcomeric and non-sarcomeric HCM triggering specific diagnostic suspicion

*Abbreviations: HCM: Hypertrophic Cardiomyopathy; TTR: Transthyretin; LVH: Left ventricular Hypertrophy; GLS: Global longitudinal strain.*


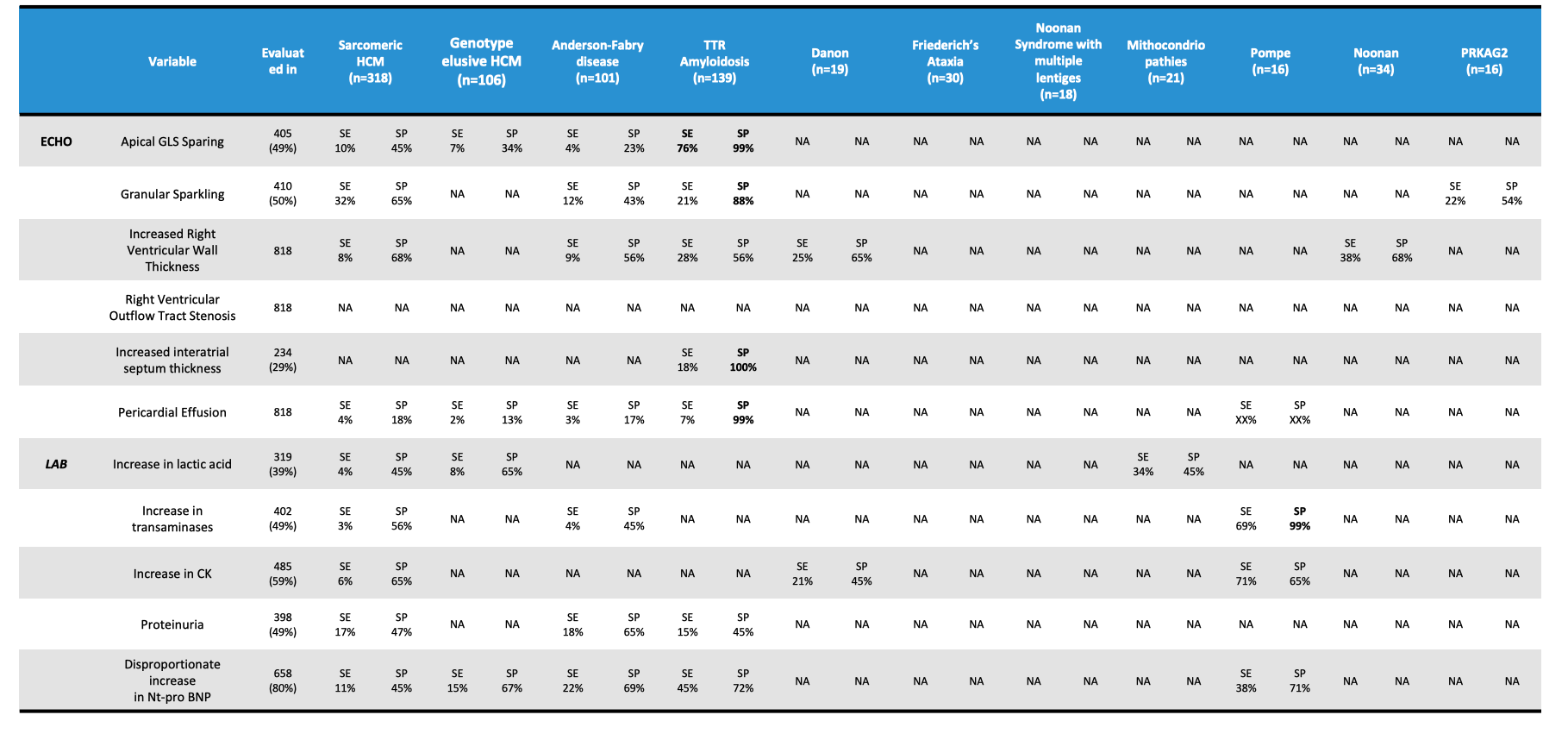


**Figure 1 Supplementary** Examples of explication windows present for each individual Red Flag detailed in the ‘Thick Heart App’

*In Panel A, the user is presented with the assessement of individual RF at ocular level; in Panel B, the explanation of biceps tendon rupture is shown; Panel C represents the guide to the assessement of giant T waves inversions on 12-lead ECG and Panel D presents a video and explanation of a typical sarcomeric HCM with an elongated anterior mitral leaflet.*


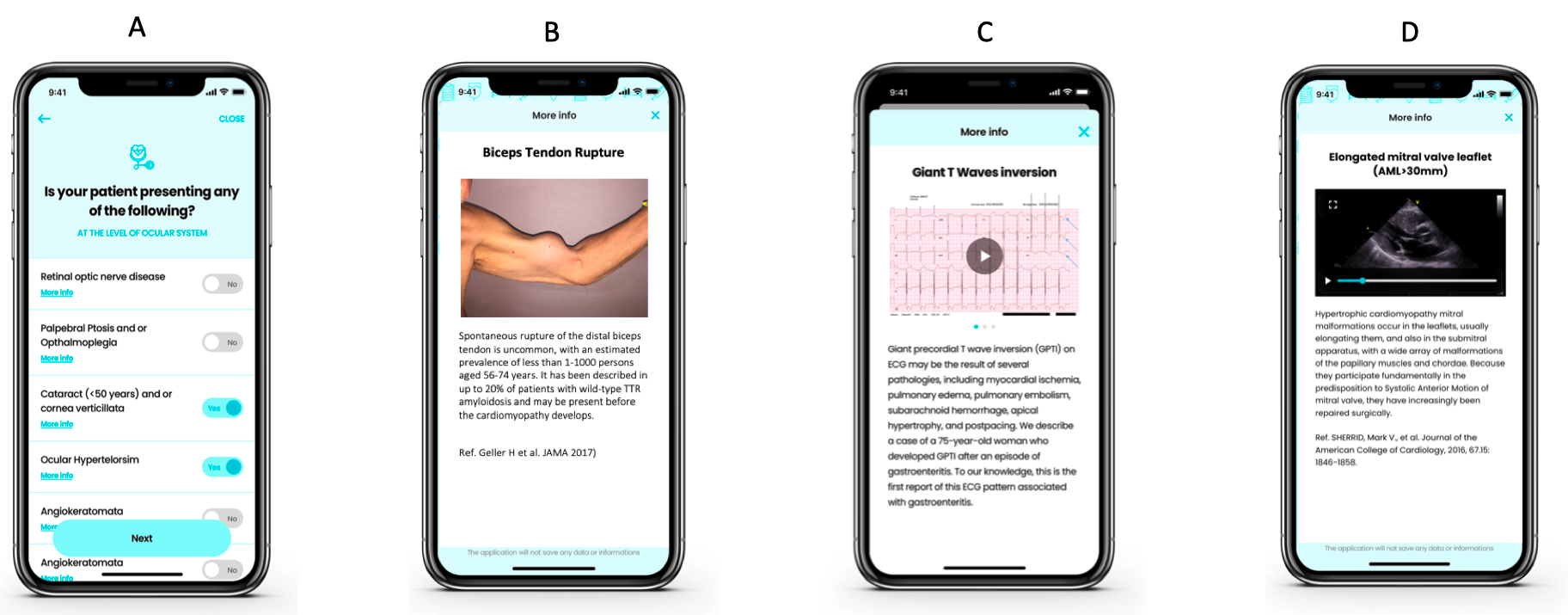

Supplement: ztaf105_Supplementary_Data [file ztaf105_supplementary_data.docx]
